# X-ray absorption spectroscopy by full-field X-ray microscopy of a thin graphite flake: Imaging and electronic structure via the carbon K-edge

**DOI:** 10.3762/bjnano.3.39

**Published:** 2012-04-25

**Authors:** Carla Bittencourt, Adam P Hitchock, Xiaoxing Ke, Gustaaf Van Tendeloo, Chris P Ewels, Peter Guttmann

**Affiliations:** 1 Electron Microscopy for Materials Science (EMAT), University of Antwerp, B-2020 Antwerp, Belgium; 2 Chemistry & Chemical Biology, McMaster University, L8S4M1 Hamilton, ON, Canada; 3 Institut des Matériaux Jean Rouxel (IMN), Université de Nantes, CNRS, Nantes, France; 4 Helmholtz-Zentrum Berlin für Materialien und Energie GmbH, Institute for Soft Matter and Functional Materials, D-12489 Berlin, Germany

**Keywords:** carbon, graphene, nanostructure, NEXAFS, X-ray microscopy

## Abstract

We demonstrate that near-edge X-ray-absorption fine-structure spectra combined with full-field transmission X-ray microscopy can be used to study the electronic structure of graphite flakes consisting of a few graphene layers. The flake was produced by exfoliation using sodium cholate and then isolated by means of density-gradient ultracentrifugation. An image sequence around the carbon K-edge, analyzed by using reference spectra for the in-plane and out-of-plane regions of the sample, is used to map and spectrally characterize the flat and folded regions of the flake. Additional spectral features in both π and σ regions are observed, which may be related to the presence of topological defects. Doping by metal impurities that were present in the original exfoliated graphite is indicated by the presence of a pre-edge signal at 284.2 eV.

## Introduction

The demonstration of the remarkable transport properties of graphene in 2004 by Geim and Novoselov triggered intense interest in its electronic structure [1–11]. A key aspect of the electronic structure, namely understanding how the graphene band structure is altered by impurity doping introduced during the synthesis, remains underexplored. Such knowledge is crucial for the reliable use of graphene in commercial electronics, for applications ranging from interconnects to high-electron-mobility transistors [12]. In graphene-based materials, the carbon atoms are assembled in a hexagonal lattice [13]. In this atomic configuration, three of the four carbon valence electrons form σ-bonds with neighbouring carbon atoms, leading to the formation of a filled σ band. The fourth electron with p_z_ symmetry, binding covalently with neighbouring carbon atoms, produces a filled π band. These occupied bands have counterparts on the unoccupied side of the Fermi edge (σ* and π* bands). In graphene sheets, the p_z_ orbital lies perpendicular to the basal plane of the planar lattice, while the sp^2^ orbitals are set in the basal plane. The high directionality of these electronic orbitals is strongly reflected in the electronic properties of graphene-based materials [14].

The near-edge X-ray-absorption fine-structure technique (NEXAFS) is ideally suited for the study of graphene-based materials, because the carbon K-edge is very sensitive to the bonding environment, providing diagnostic information about the structure, defects and doping [15]. Photoabsorption of X-rays creates core-level excited states in which a 1s electron is promoted into the unoccupied π* or σ* bands. In a planar system, such as graphene, there is a strong selection rule for this process with (1s^−1^, π*) final states being selectively excited when the *E*-vector lies perpendicular to the basal plane, and (1s^−1^, σ*) final states being selectively excited when the photon *E*-vector lies in the basal plane. The presence of defects, nonplanarity and edges relax these symmetry selection rules. The peak energy positions and line shapes of the measured NEXAFS resonances reflect the unoccupied density of states, although the energies and intensities are modified by core–hole interactions [15]. When linearly polarized X-rays are used the carbon K-edge of graphene materials exhibits a strong linear dichroism, which can be used to probe local anisotropy and structural order [16]. When combined with X-ray microscopy, NEXAFS can be used to study isolated, free-standing nanostructures [17–18]. Linear dichroism can be used to filter the signal according to selection rules based on the symmetry of the sample electronic states and orientation with respect to the polarisation of the *E*-vector. Deviation from the expected signal intensity can be associated with nonplanarity, structural defects, etc. [12].

Here we use NEXAFS spectromicroscopy, performed with the Helmholtz Zentrum Berlin (HZB) full-field transmission X-ray microscope (TXM) installed at the electron storage ring BESSY II [19], to image and to study the electronic structure of a free-standing thin graphite flake produced by means of density-gradient ultracentrifugation (DGU) [20]. In the DGU process the bile salt sodium cholate (C_24_H_39_O_5_Na) is used to promote graphite exfoliation, resulting in graphene–surfactant complexes having buoyant densities, which vary with the graphene thickness. Raman studies on samples produced by the same technique found no impurities, such as amorphous carbon, in the sample [21–22].

**Figure 1:**
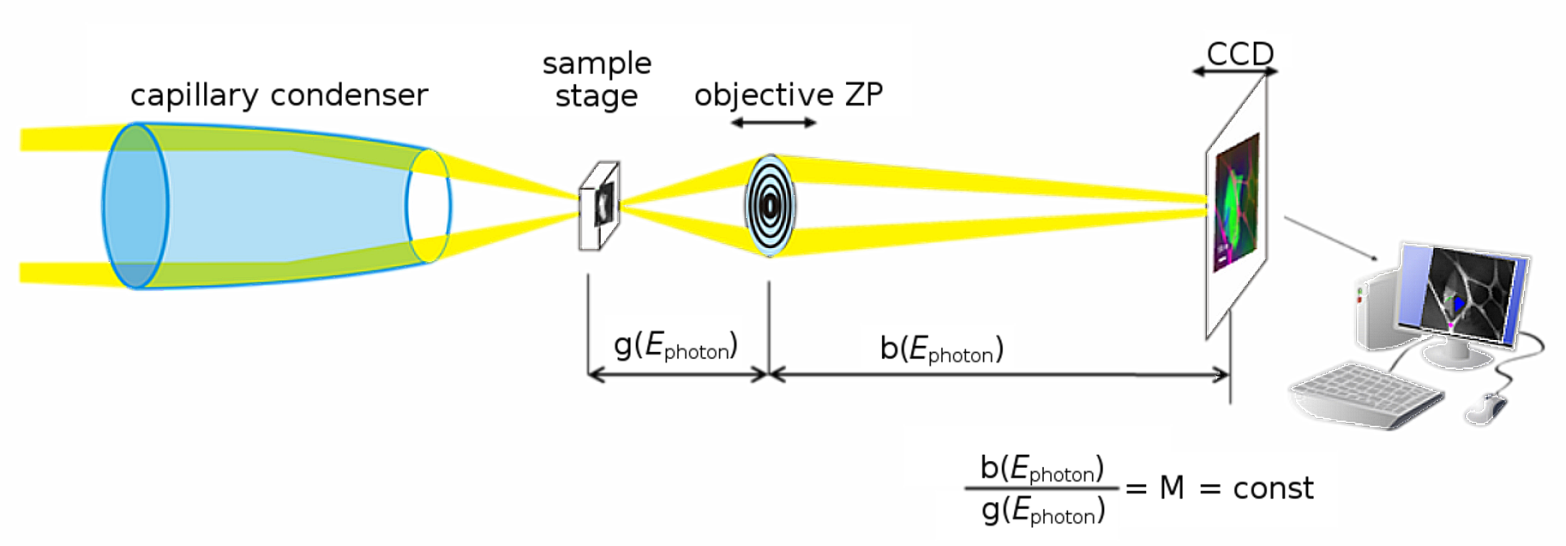
Schematic of the TXM for NEXAFS studies. Monochromated radiation from the undulator is focused by a reflective capillary condenser into the object field. A zone plate objective forms a magnified image on the CCD.

The setup of the HZB full-field X-ray microscope (Figure 1) is analogous to that of a bright-field light microscope: the sample is illuminated by quasi-monochromated X-rays by using a reflective capillary optic as a condenser [23]. The sample is imaged by a zone plate objective with a CCD camera. With a field of view of 15–20 μm (diameter) several nanostructures can be imaged in the same image or image sequence (stack). During the data acquisition for a NEXAFS image stack, only the objective is moved, since the reflective condenser works largely independently of the photon energy of the illuminating X-rays. For the different photon energies there are slight changes in magnification, which are corrected by relocation of the CCD camera (Figure 1) [19]. Polarization (linear dichroism) studies are performed by rotating the sample relative to the orientation of the fixed polarization vector of the incident photons.

## Results and Discussion

**Figure 2:**
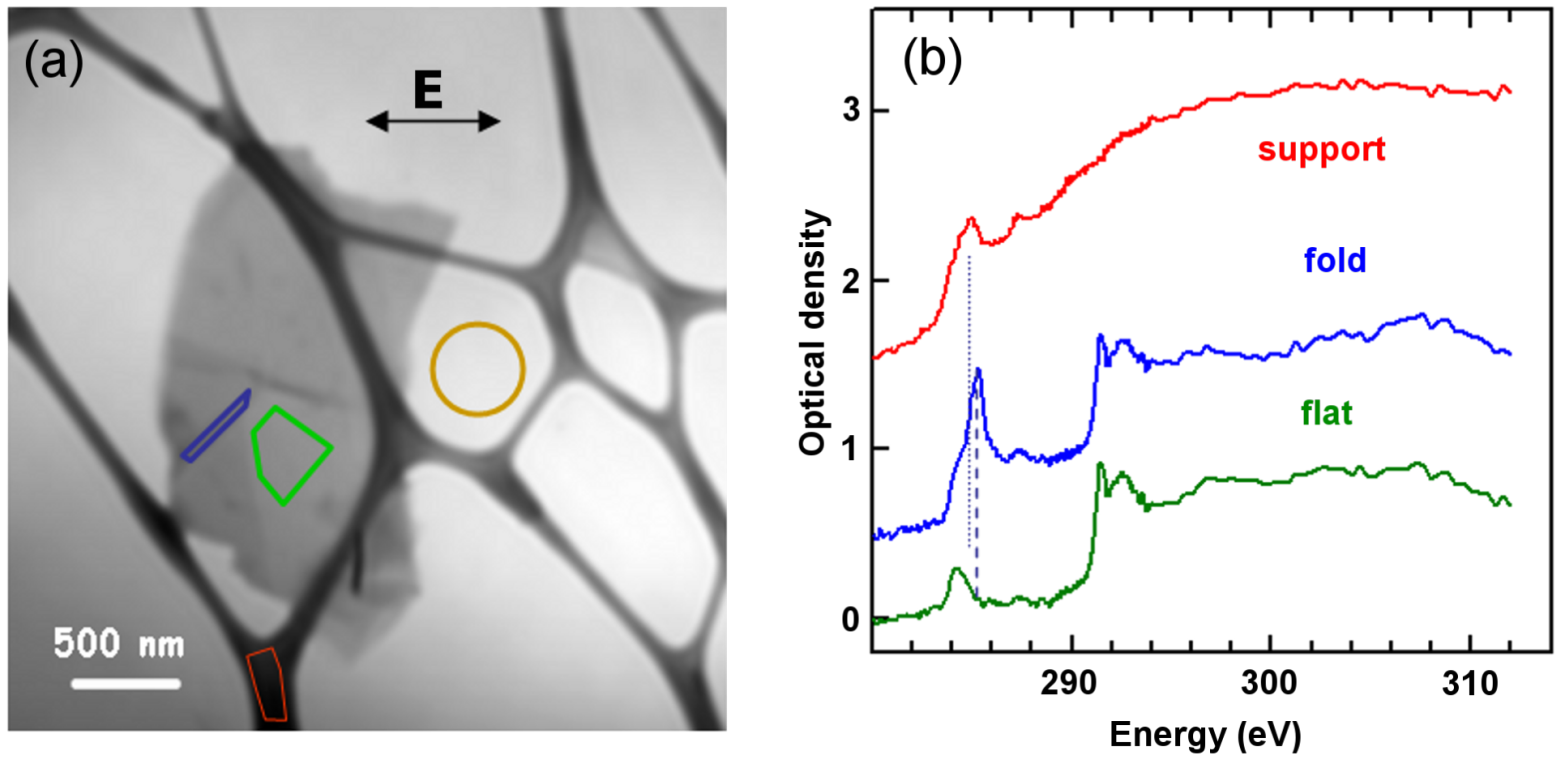
(a) Average of aligned TXM images (283–311 eV). (b) Spectra extracted from the three regions indicated by the coloured shapes in part (a): red is from the lacey carbon support, green is from the flat area of the sample, while blue is from the folded region. The signal intensity used to convert the measurements to optical density (OD) was taken from the yellow circular area. Offsets of 0.5 (blue) and 1.5 (red) OD have been used for clarity.

We examined several different flakes to ensure that we had selected a typical specimen; electron microscopy images are given in Supporting Information File 1. The following analysis concerns the single flake shown in Figure 2a. This sample has an average thickness of ~100 nm. Generally, optimal samples for carbon K-edge TXM have an optical density (OD) around 1, which for multilayered graphene samples corresponds to about this thickness. In principle, absorption signals detected by transmission are observable down to an OD of 0.01, which would correspond to a multilayered graphene sample of three layers thick. Even-thinner samples represent a significant instrumental challenge, but with sufficient stability and signal integration it should be possible to measure the carbon K-edge NEXAFS of a single layer of graphene. The thickness of the studied sample ensures a good signal-to-noise ratio allowing detailed analysis of the peak form and composition.

Figure 2a shows an average of 236 TXM transmission images (after alignment), from 283 to 311 eV. From the differences in contrast we can identify areas with a different number of sheets, and also some steps and some folds (schematically marked in Supporting Information File 1). Figure 2b presents C 1s spectra from selected regions, indicated by the coloured shapes in Figure 2a. The most intense peak in the C 1s NEXAFS spectra at 285.3 eV is due to electronic excitation from the C 1s level to the conduction π* states. This transition should be forbidden by selection rules, in the geometry used (**E** parallel to the basal plane). However, we see a residual intensity due to the folding of a few graphene layers, implying that there is a nonzero angle between the polarization vector and the basal plane in places. This is most notable in the central spectrum, confirming that this region corresponds to a fold in the flake.

The peak near 292 eV corresponds to the σ* threshold. This peak is composed of two distinct features at 291.5 eV and 292.6 eV. The sharp feature at 291.5 eV is an exciton [24], its sharpness reflecting the strong correlation effects of electron–hole pairs within the flake. The broad peak at 292.6 eV is related to the transition from the C 1s level to the relatively nondispersing σ* states at the Γ point of the Brillouin zone (BZ) [25].

For the HZB TXM the *E*-vector of the light is always in the horizontal plane, which is thus in the plane of the graphene sheets for the unfolded area, but approximately perpendicular to the folds. Thus the blue spectrum (Figure 2b) of the fold exhibits a strong C 1s → π* peak at 285.3 eV, since the *E*-vector is orthogonal to the graphene sheets folded out of the plane of the sample. In contrast, the signal at the corresponding transition energy in the flat part of the graphene sample is negligible, as expected when the *E*-vector is in the plane of the sheets. In the spectrum recorded in the flat region of the sample, the peak near 285.3 eV has much lower intensity than in the folded region, because the angle between the polarization vector and the basal plane is nearly zero.

The peak at 284.2 eV, very clear in the spectrum of the flat part of the graphene, also exists at about the same intensity relative to the C 1s continuum intensity, in the spectrum of the fold. Schultz et al. [12] assigned this pre-edge structure to dopant-induced states. As we did not observe this in the spectrum recorded on an amorphous carbon film with the sodium cholate, we suggest that this structure arises instead from metal impurities in the graphite used for exfoliation. A careful examination of the spectrum of the folded region (Figure 2) shows the presence of a shoulder at the same photon energy of this structure, indicating that the doping is rather uniform in the flake.

In a recent theoretical study, spectral features appearing at 287–290 eV photon energy were associated with topological defects, such as monovacancies, divacancies and Stone–Wales defects [26]. In particular, a structure centred at 287 eV was assigned to carbon atoms beside divacancy defects [26]. Accordingly, the structure observed at 287.4 eV in the NEXAFS spectra recorded in both the flat and folded regions may be thus associated.

**Figure 3:**
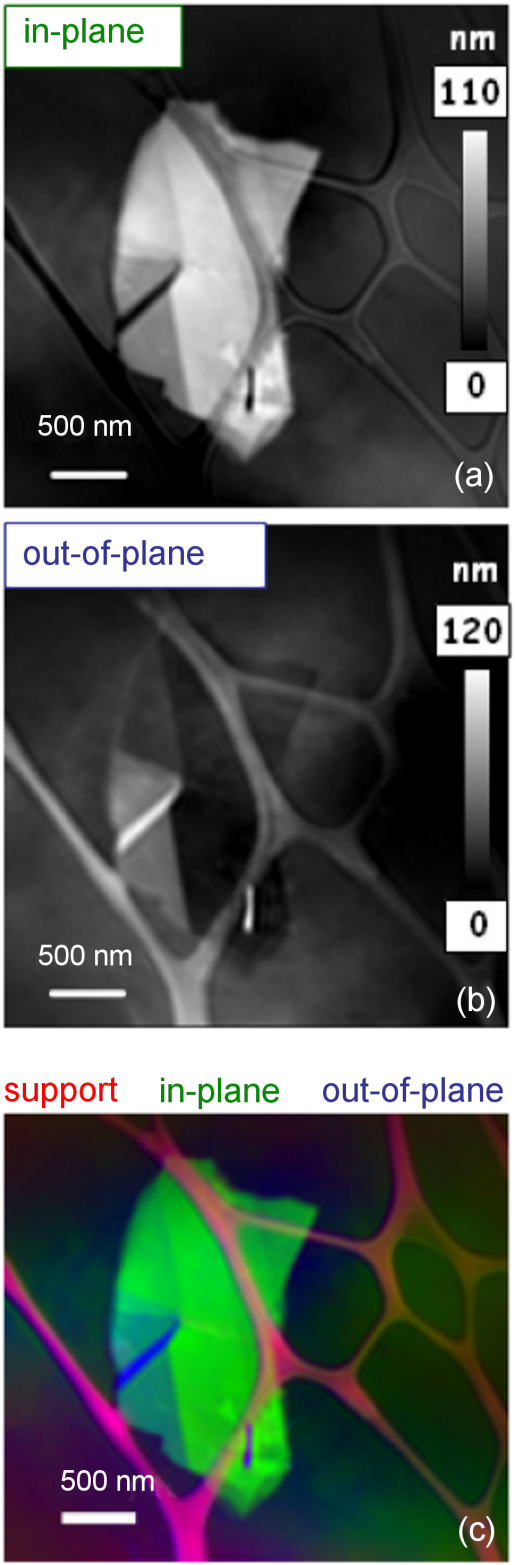
Component map of (a) in-plane (σ) and (b) out-of-plane (π) bonds; (c) colour-coded composite of the maps for lacey carbon (red), in-plane (green) and out-of-plane (blue), derived from a three-component fit of the carbon K-edge image sequence.

The spectra in Figure 2b can be used as reference signals to discriminate, in the X-ray image of the sample, between the regions that are in-plane (flat regions), out-of-plane (folded region) and the lacey carbon. Figure 3 presents results from a three-component fit by using the spectra shown in Figure 2b as reference signals. In the images, flat and folded regions can be easily distinguished, as can step edges. In the in-plane mapping (σ*) the folded region appears black, i.e., the (π*) intensity is minimum while in the out-of-plane region it is maximum. At the edges of a graphene sheet the π orbitals are tilted, thus a marked increase/decrease in the π* intensity relative to σ* in the in-plane/out-of-plane component map is expected. Combining the three images in Figure 3, the morphology of the sample can be well described (Figure S1, Supporting Information File 1).

We also scanned over the O 1s spectral range 528–556 eV, and the absence of signal confirms that the sample is essentially oxygen free (Figure S2, Supporting Information File 1). In addition this confirms the absence of sodium cholate (C_24_H_39_O_5_Na), which could appear, for example, due to residual intercalation in the flake.

In summary, the distinctly different X-ray spectra of the flat and folded regions of the sample, which are related to the strong dichroism of the C 1s spectrum of graphene, have been used in a fitting procedure to generate maps of the flat and folded regions of the flake. A low-lying peak at 284.2 eV is associated with doping states in the electronic structure of the flake induced by metallic impurities present in the graphite that was exfoliated to make the multilayer graphene sample. We tentatively associate a peak at 287.4eV with defects created during the exfoliation using sodium cholate (C_24_H_39_O_5_Na).

The combination of TXM with NEXAFS has the potential to become an important tool in nanotechnology, particularly in the imaging and analysis of nanoscale samples that are sensitive to electron irradiation. The current study shows that it is applicable to the study of carbon nanomaterials, notably large two-dimensional structures for which statistical spectroscopic analyses over large areas are to be preferred.

## Experimental

The carbon K-edge images were recorded with the TXM installed at the undulator beamline U41-FSGM at the electron storage ring BESSY II of the HZB, Berlin, which has a spatial resolution of up to 11 nm and a spectral resolution up to *E/∆E ≈* 10000. Images were recorded at room temperature in transmission mode over a sequence of photon energies from 280 eV to 312 eV, with a spectral resolution *E*/Δ*E* ≥ 4500. The exit slit of the monochromator was set to 5 µm, which corresponds to a calculated spectral resolution of *E*/∆*E* = 20000.

The data analysis was accomplished by using aXis2000 [27]. The NEXAFS spectra were normalized by using the signal intensity of the sample (the circled area in Figure 2) to correct for variations of the photon flux with photon energy (*h*ν) and acquisition time.

The sample was obtained from NanoIntegris (Illinois, USA) in the form of “PureSheets” (MONO). The aqueous solution (0.5 μg per 10 μL) with an ionic surfactant (2% w/v) contains pristine multistacked graphene flakes with different numbers of sheets, which have not been oxidised, reduced, or chemically modified in any way.

## Supporting Information

Schematic of the morphology of the studied sample, electron microscopy images of typical samples and oxygen K-edge spectrum.

File 1 Additional figures.
