# Supplementary material for: X-ray absorption spectroscopy by full-field X-ray microscopy of a thin graphite flake: Imaging and electronic structure via the carbon K-edge
Source: Beilstein J Nanotechnol. 2012 Apr 25;3:345–50. doi: 10.3762/bjnano.3.39 (PMC3388357; doi:10.3762/bjnano.3.39)
Supplement: File 1 — Additional figures. [file Beilstein_J_Nanotechnol-03-345-s001.pdf]

Supporting Information

for

X-ray absorption spectroscopy by full-field X-ray microscopy of a thin graphite flake: Imaging and electronic structure via the carbon K-edge.

C. Bittencourt*[1], A. P. Hitchock[2], X. Ke[1], G. Van Tendeloo[1], C. P. Ewels[3] and P. Guttmann[4]

Address: [1]Electron Microscopy for Materials Science (EMAT), University of Antwerp, B-2020 Antwerp, Belgium, [2]Chemistry & Chemical Biology, McMaster University, L8S4M1 Hamilton, ON, Canada, [3]Institut des Matériaux Jean Rouxel (IMN), Université de Nantes, CNRS, Nantes, France and [4]Helmholtz-Zentrum Berlin für Materialien und Energie GmbH, Institute for Soft Matter and Functional Materials, D-12489 Berlin, Germany

Email: Carla Bittencourt* - carla.bittencourt@ua.ac.be

* Corresponding author

Additional figures

Figure S1a shows an electron microscopy image recorded on typical flakes studied in this work. We can see the morphology of a typical edge with a few layers in Figure S1b.

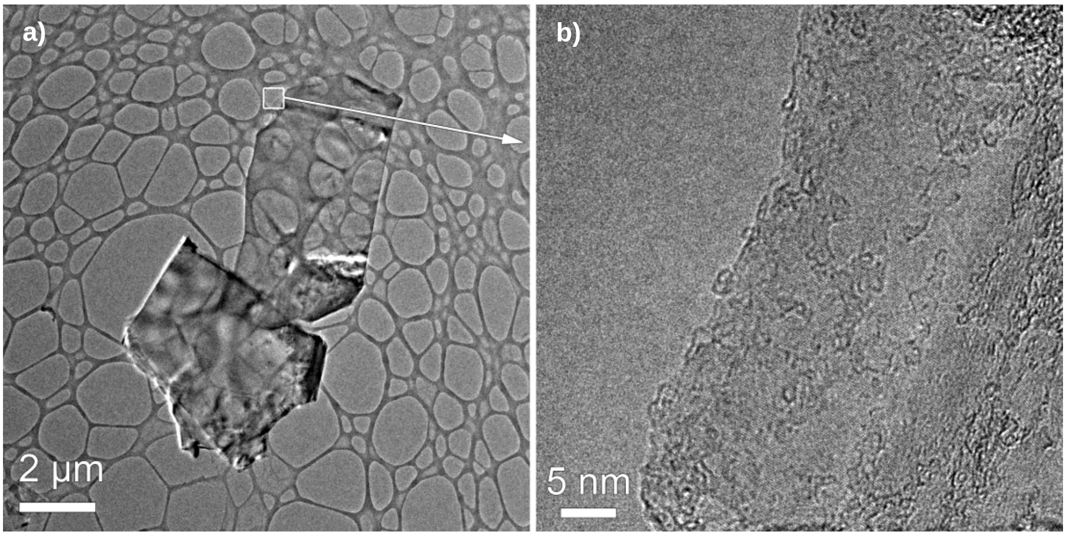

Figure S1: Typical flakes: Electron microscopy image at low (a) and high (b) resolution.

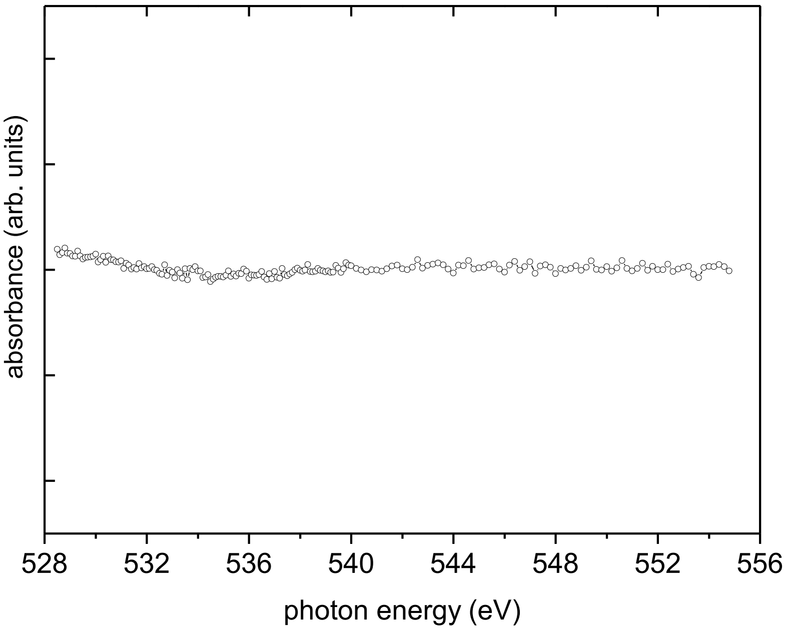

Figure S2: Oxygen K-edge spectrum

Figure S3 shows the schematic of the morphology of the studied flake. Differences in the contrast level in the X-ray image (see Figure 3) allow the determination of regions with different OD (optical density) and edges.

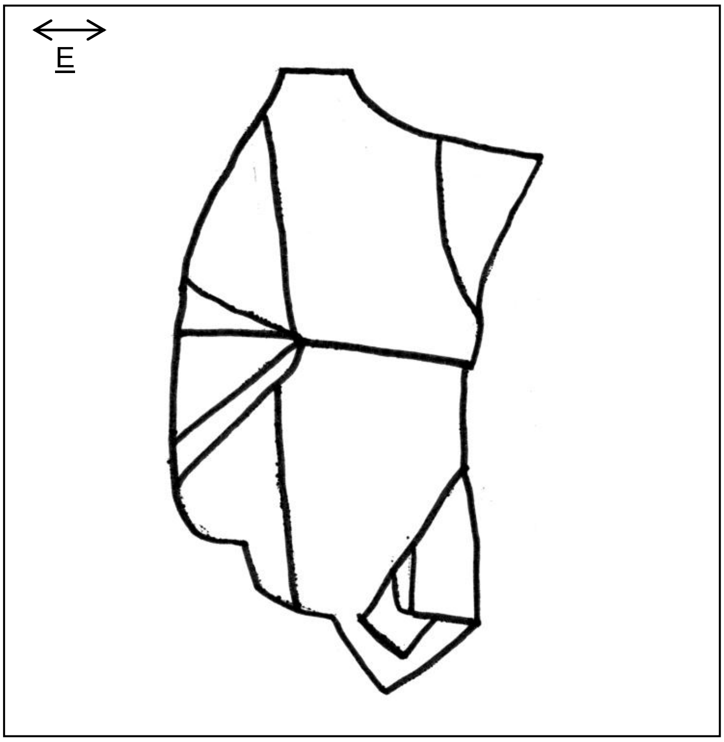

Figure S3: Schema of the morphology of the studied sample. The images in Figure 3 were used to determine the different edge locations.
